# Immunotoxicity of Inhalable Organic Dust Samples Based on In Vitro Analysis of Human Respiratory Epithelial Cells

**DOI:** 10.3390/ijms27031433

**Published:** 2026-01-31

**Authors:** Marcin Cyprowski, Lidia Zapór, Aneta Ptak-Chmielewska, Paweł Kozikowski

**Affiliations:** 1 Laboratory of Biohazards, Central Institute for Labour Protection—National Research Institute, 00-701 Warsaw, Poland; 2 Laboratory of Toxicology, Central Institute for Labour Protection—National Research Institute, 00-701 Warsaw, Poland; lizap@ciop.pl; 3 Institute of Statistics and Demography, Warsaw School of Economics, 02-554 Warsaw, Poland; aptak@sgh.waw.pl; 4 Laboratory of Electron Microscopy, Central Institute for Labour Protection—National Research Institute, 00-701 Warsaw, Poland; kozz84@gmail.com

**Keywords:** inhalable organic dust, SEM analysis, workers’ exposure, respirable crystalline silica, bioaerosols, human respiratory epithelial cells, cytotoxicity, proinflammatory cytokines, linear ordering

## Abstract

Airborne organic dust has rarely been subject to immunotoxicological analysis. A pilot study was undertaken to link exposure metrics (respirable crystalline silica (RCS), bacteria, fungi, endotoxins (END), peptidoglycans (PGN), (1 → 3)-β-D-glucans (GLU)) with in vitro cytotoxicity and cytokine responses based on analysis of airborne organic dust samples collected during a single work shift at six different facilities. The A549 and BEAS-2B cell lines were used to assess cytotoxicity and proinflammatory cytokine release. The general linear model (GLM) and taxonomic linear ordering were used to identify key determinants and rank facilities by the hazard level they pose. The highest cytotoxicity of organic dust was observed at the sewage treatment plant, while the lowest was at the poultry farm. The most hazardous agents present in organic dust included RCS, aerobic bacteria, fungi, PGN, and GLU. They significantly affected cytokine release, particularly of IL-6 and IL-8. The use of a synthetic measure showed that inhalable organic dust from the composting plant presented the highest potential to induce adverse effects on human health, while the lowest one was characterized by the biomass-fired power plant samples. The open-ended statistical method can significantly increase awareness of occupational hazards and promote more responsible protection for exposed workers.

## 1. Introduction

Organic dust can be defined as a heterogeneous mixture of non-biological particles (e.g., respirable crystalline silica) and biological particles of plant, animal, and micro-biological origin. The microbial components comprise both viable and non-viable particulate matter, such as bacteria, fungi, and viruses, as well as their toxins and metabolites, including endotoxins, peptidoglycans, (1 → 3)-β-D-glucans, and mycotoxins [1]. It is difficult to precisely determine the composition of organic dust as it is influenced by a variety of factors, including the environment of origin, microclimatic conditions, and seasonal variability of its content [2,3,4].

At first, the formation of organic dust was associated with the working conditions of farmers and cotton growers. However, the number of jobs and industrial settings in which its presence has been confirmed is much larger. These include, e.g., intensive plant and animal production [5], municipal waste management [6], and sewage treatment [7]. Moreover, organic dust can be found in the plant processing industry [8], particularly in facilities where biomass is processed for energy generation [9]. Organic dust exposure also affects the workers at archive storage sites and libraries [10] and office workers [11]. It is estimated that several hundred million people worldwide may be exposed to organic dust during their occupational activities. In the United States alone, in the year 2000, the number of workers occupationally exposed to grain dust reached 0.5 million, and to cotton dust, 0.8 million [12]. In India, recent estimates show that more than 20 million people employed in intensive livestock production may be exposed to organic dust [13].

Inhalation of organic dust particles usually results in respiratory symptoms, including irritation of the upper and lower respiratory tract, manifested by a runny nose, chronic cough, shortness of breath or wheezing, and chest tightness [14,15,16,17]. Furthermore, exposure to organic dust may lead to various diseases of allergic or immunotoxic etiology, such as hypersensitivity pneumonitis (HP), organic dust toxic syndrome (ODTS), chronic obstructive pulmonary disease (COPD), and asthma [18,19,20,21]. However, confirming the diagnosis of these non-infectious diseases can be challenging, as employees may be reluctant to seek medical advice or fail to report the symptoms for fear of losing their jobs [22]. The result of this practice is the so-called “healthy worker effect” and symptom underreporting [23].

A possible reason why the problem of organic dust exposure and its potential health effects has not been adequately considered could be a fallacious methodology in relevant epidemiological studies [24], as well as restrictions on environmental studies during the COVID-19 pandemic [10]. These impediments have made researchers seek alternative methods to study the impact of organic dust on human health. One such method, to be performed under laboratory conditions, is the use of cell lines to investigate the mechanisms and pathways underlying the effects of such exposure on the respiratory system, including interactions between exposure agents. The major area of interest is the use of the A549 line (human non-small-cell lung cancer cell line), whose properties resemble those of type II alveolar cells, making it an attractive research model for studying the impact of organic dust components on lung epithelium [25,26,27,28,29]. Other possible cell lines to be used for this purpose include the immortalized human bronchial epithelial cell line (BEAS-2B) and human monocytic cell line (THP-1) [30,31]. In experimental studies, the respiratory tract is typically represented by bronchial epithelial cells (BEAS-2B). Thanks to their ability to form tight junctions and to adhere “foreign” particles on the surface of cell membranes, BEAS-2B cells are the body’s first line of defense against the penetration of such particles into cells [32]. They are considered a sensitive model for toxicity testing of environmental pollutants, including those present in organic dust [33,34].

In studies conducted so far, the most frequently analyzed samples were those of the settled dust from various work environments. In contrast, airborne organic dust has rarely been subject to such analysis [29,30,35,36]. This seems surprising, since measuring airborne dust concentrations in workplaces is part of industrial hygiene routines, and the samples could be used for more specific analysis. One possible testing direction for dust samples collected during a single work shift could be the immunotoxicity of organic dust to human respiratory epithelial cells. To summarize the facts described above, there is still a lack of integrated airborne dust-immunotoxicity-hazard ranking studies. Furthermore, organic dust from some facilities may affect higher cytotoxicity and proinflammatory responses, suggesting that specific micro-biological or inorganic components may be key determinants.

As we believe the immunotoxicity assay is essential for an overall assessment of health effects of organic dust exposure, we have undertaken a pilot study to investigate the immunotoxic properties of organic dust, based on analysis of air samples collected during a single work shift at six different facilities. Assessment of exposure to inhalable organic dust included respirable crystalline silica (RCS), aerobic and anaerobic bacteria, fungi, endotoxins (END), (1 → 3)-β-D-glucans (GLU), and peptidoglycans (PGN). The A549 and BEAS-2B cell lines were used to assess cytotoxicity and proinflammatory cytokine release in respiratory epithelial cells exposed to organic dust extracts.

To estimate potential health hazards for workers exposed to inhalable organic dust, the collected data were subjected to a multifactorial analysis, and synthetic measures were then used to rank the organic dusts by the level of hazard they pose.

## 2. Results

### 2.1. Organic Dust Surface Morphology

**Figure 1:**
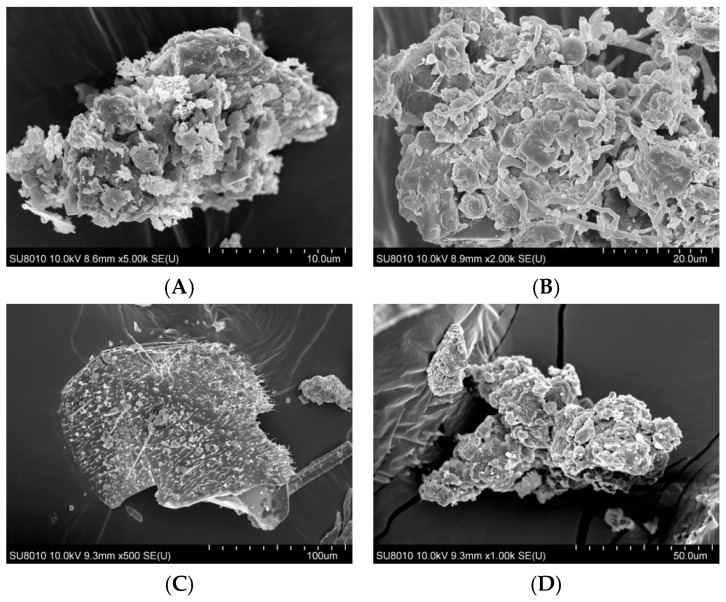

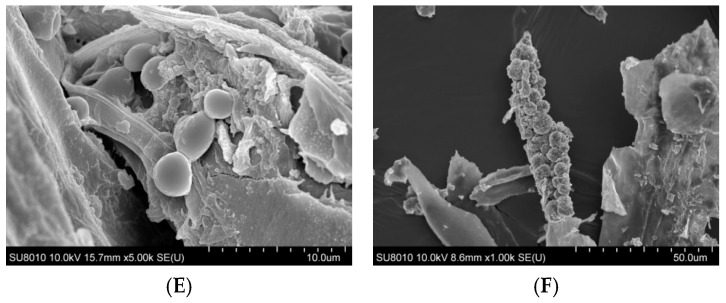
SEM images of inhalable organic dust samples ((**A**)—WSP, (**B**)—WCP, (**C**)—STP, (**D**)—CP, (**E**)—PP, (**F**)—PF).

The surface morphology of organic dust particles, assessed by Scanning Electron Microscope (SEM), varied depending on their source (Figure 1A–F). The particles collected from a waste sorting plant (WSP) and a waste composting plant (WCP) had irregular shapes and rough, porous surfaces while the majority of particles originating from a cement plant (CP) and a power plant (PP) presented a more compact and smooth surface structure. In contrast, the samples collected from a poultry farm (PF) showed the presence of particles with spherical structures, which points to their biological origin. Energy Dispersive Spectroscopy (EDS) analysis revealed that the dust samples had varying contents of chemical elements (Table 1).

EDS analysis indicated qualitative differences in elemental surface composition between dust samples from different facilities (Table 1). Dust samples from the WSP and the sewage treatment plant (STP) showed the presence of a broader range of detectable elements, including several inorganic ones, whereas samples from the PF and the WCP were characterized by a more limited set of detected elements. The presence of carbon was detected in all samples; however, it should be noted that EDS has limited reliability for qualitative or quantitative assessment of light elements such as carbon, nitrogen, and oxygen. In addition, the use of carbon adhesive tapes for sample mounting may contribute to surface carbon signals, further limiting interpretation of light-element content. Potential carbon contamination may result from using carbon tape for quick sample mounting for EDS applications. Unfortunately, the technique used does not allow unambiguous identification of the chemical compounds forming organic dust, since EDS provides only elemental information on organic compounds and cannot determine their molecular structure. We presume these could have been various types of amino acids, indicating the presence of organic matter. However, the dust samples could also contain complex organic compounds, such as microplastic particles. The oxygen and nitrogen atoms identified on the organic dust surface were not present in a gaseous form. However, their detection indicates the presence of various oxides (e.g., silicon, aluminum, calcium oxides) and of organic and inorganic nitrogen compounds.

### 2.2. Exposure Assessment

Measurements of inhalable organic dust enabled determination of exposure levels to microbial components during a single work shift in particular plants. Detailed results of the analyses are presented in Table 2.

They demonstrated that the highest mean inhalable dust concentrations were recorded at workstations in the PP. The highest concentrations of RCS and fungi were detected in the WSP, while those of PGN and GLU were detected in the WCP workstations. Furthermore, aerobic and anaerobic bacteria, as well as END, were found in the PF. For all microbial agents, differences between facilities were statistically significant. Among other findings, a strong correlation was noted between aerobic and anaerobic bacteria in inhalable organic dust (r = 0.82; *p* < 0.001). Moderate correlations were found between dust concentration and the concentrations of RCS and END, those of RCS and fungi, as well as END and anaerobic bacteria (Table 3).

### 2.3. Cytotoxicity Assessment

Exposure measurements set up a starting point for immunotoxicity studies. For this purpose, the concentrations of individual exposure agents were converted into one milligram of dust to prepare suspensions for exposure of the A549 or BEAS-2B cell lines (Table 4). This transformation of the measurement data revealed that the concentrations of some exposure agents, in 1 mg of organic dust, differed from those determined directly in workplace air (Table 2). This referred particularly to RCS, the highest concentration of which was found in STP samples; to fungi in WCP; and to END in WSP. Furthermore, for most of the analyzed agents, significant differences between the facilities were again demonstrated, except for END, whose content was at levels that were statistically comparable.

**Figure 2:**
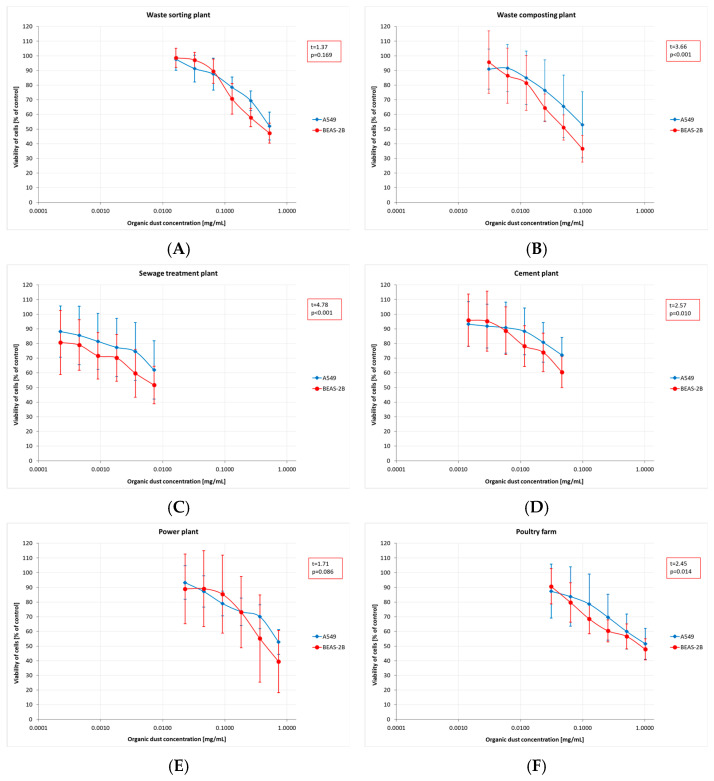
Cytotoxicity assessment (by MTT test) of inhalable organic dust samples from the facilities under study (AM (SD)). Data are expressed as the percentage viability of cells exposed to dusts relative to control cells. Each point represents data from at least two independent experiments. (**A**)—waste sorting plant; (**B**)—waste composting plant; (**C**)—sewage treatment plant; (**D**)—cement plant; (**E**)—power plant; (**F**)—poultry farm.

The results of toxicity assessment, displayed in Figure 2A–F, are presented as graphs showing a decline in average cell viability. Cytotoxicity assays revealed a decrease in cell viability, irrespective of dust concentration and sampling site. Furthermore, the magnitude of the decrease differed between the cell lines used. BEAS-2B cells proved to be significantly more sensitive, with an average viability of 2–9 percentage points lower than that of A549 cells. For most of the plants studied, these differences were statistically significant, except for the dust samples collected at the WSP.

**Figure 3:**
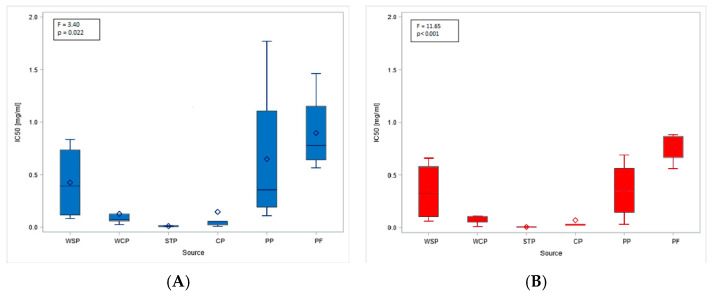
IC50 values for inhalable organic dust samples from facilities under study, based on the study of A549 (**A**) and BEAS-2B (**B**) cell lines. Rhombuses—arithmetic means, solid lines inside boxes—median values, boxes—1st and 3rd quartiles, whiskers—range of values. WSP—waste sorting plant; WCP—waste composting plant; STP—sewage treatment plant; CP—cement plant; PP—power plant; PF—poultry farm.

**Figure 4:**
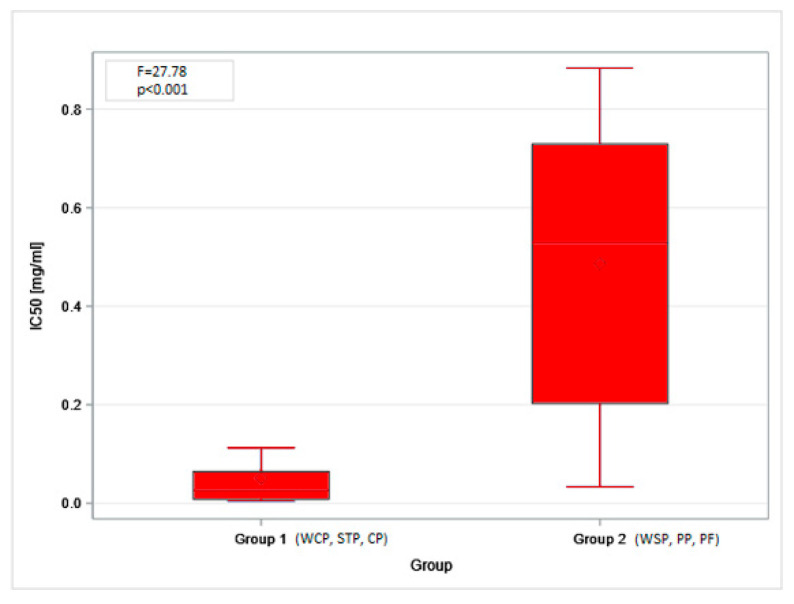
IC50 values for inhalable organic dust samples classified according to the cytotoxic effect on the BEAS-2B cells. Rhombuses—arithmetic means, solid lines inside boxes—median values, boxes—1st and 3rd quartiles, whiskers—range of values. WSP—waste sorting plant; WCP—waste composting plant; STP—sewage treatment plant; CP—cement plant; PP—power plant; PF—poultry farm.

The obtained MTT (Methyl Thiazolyl Tetrazolium) test results were directly translated into the IC20 and IC50 indices. The highest mean IC20 value (0.17 mg/mL, SD = 0.21) was obtained in the PF. However, these concentrations could not be determined for the dust samples from the STP and CP. The differences in low-toxicity concentrations (IC20 values) across facilities (regardless of the cell type tested) were not statistically significant. In contrast, IC50 values showed statistically significant differences depending on the sampling site. The lowest mean values, indicating the highest cytotoxicity of organic dust, were found in the STP (0.01 mg/mL, SD = 0.01), while the highest ones were in the PF: 0.77 mg/mL (SD = 0.14) and 0.90 mg/mL (SD = 0.39), for BEAS-2B and A549 cells, respectively (Figure 3A,B). The findings on cytotoxicity levels enabled the distinction of two groups of organic dust samples that differed in their effects on the cell lines. The samples from the WCP, STP, and CP showed significantly higher cytotoxic activity on BEAS-2B cells than the dust samples from the WSP, PP, and PF, as shown in Figure 4.

Previously prepared organic dust suspensions were used to determine the level of proinflammatory cytokines released by the cell lines. The cytokine secretion profile of the two cell lines differed depending on the cytokine tested. TNF-α was released at the lowest amount, with most of the concentrations being below the detection level. For A549 cells, the percentage of positive results was 25%, whereas for BEAS-2B cells, it was only 15%. Therefore, TNF-α was not included in further analyses. IL-1β was released at a significantly higher rate by BEAS-2B cells (65% positive results) than by A549 cells (13%), so only the concentration values referring to BEAS-2B were considered. For IL-6 and IL-8, the percentage of positive results for both cell lines was 62–65%, which allowed for including them in further analyses.

**Figure 5:**
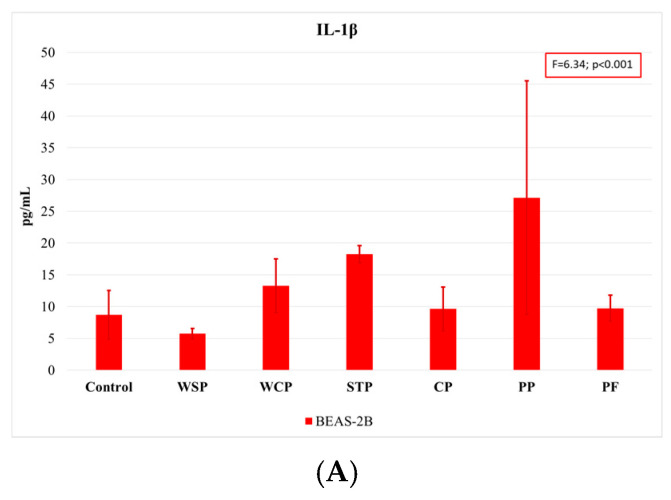

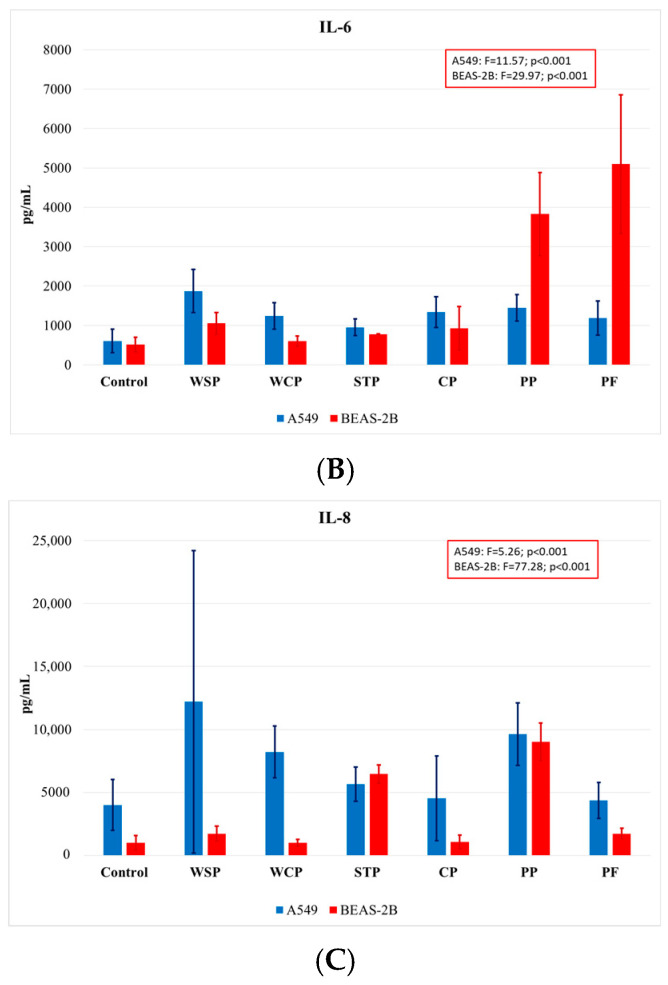
Average concentrations of proinflammatory cytokines IL-1β (**A**), IL-6 (**B**), and IL-8 (**C**) released by A549 and BEAS-2B cell lines, as analyzed by facilities under study (AM (SD)). WSP—waste sorting plant; WCP—waste composting plant; STP—sewage treatment plant; CP—cement plant; PP—power plant; PF—poultry farm.

Comparing the findings on cytokine concentrations across facilities, one can note that the highest rates of IL-1β release were observed when cells were exposed to dust samples from PP, and the lowest ones when they were exposed to those from WSP (Figure 5A). The highest IL-6 concentrations (Figure 5B) were measured in BEAS-2B cells after exposure to dust samples from PF and PP, while the lowest levels after exposure to samples from the WCP and STP. With regard to IL-8 release (Figure 5C), the highest concentrations were observed in the A549 cells after exposure to dust samples from the WSP, and the lowest ones after exposure to dust from the CP.

Table 5 presents Pearson correlation coefficients for the cytotoxicity assessment results and cytokine concentrations. A strong, significant correlation was found only for IC50 and IL-6 released by the BEAS-2B cells.

A generalized linear model (GLM) was used to identify exposure agents that significantly affected the release of individual cytokines by particular cell lines. Gamma distribution with log-link function was used in GLM model for IL-8 and normal distribution with identity-link function for IL-6 in A549 modeling. Gamma distribution with log-link function was used in the GLM model for IL-8 and Gamma distribution with identity-link function for IL-6 and Gamma distribution with log-link for IL-1B in BEAS-2B modeling. Selection of model was based on best goodness of fit to the data. Data were not transformed prior to modeling and we did not exclude correlated predictors to avoid losing too much information. In the case of correlated predictors, the estimates are consistent but not efficient (high variance), only for correlated variables. The results obtained for the A549 cell line are presented in Table 6 and for the BEAS-2B line in Table 7. After examining dust samples from the six facilities, we found that the factors having the strongest impact on IL-1β cytokine release by BEAS-2B were the concentrations of inhalable organic dust, in particular, the PGN concentrations. The factors that had a significant influence on IL-6 release included the concentration levels of inhalable dust, RCS, fungi, and aerobic bacteria (for both cell lines). The IL-8 concentrations were significantly affected by the following agents: RCS, fungi and aerobic bacteria (both cell lines), END, PGN, and GLU (BEAS-2B).

### 2.4. Linear Ordering

The data on exposure level, cytotoxicity of organic dust, and concentrations of proinflammatory cytokines released by the cell lines used in this study served as a starting point for assessing the potential health hazards from exposure to inhalable organic dust in particular facilities and for presenting it in the form of a hazard ordering chart. Taxonomic linear ordering was used to calculate synthetic variables and to evaluate health hazards from organic dust exposure (Table 8). The following variables were used as destimulants: dust [mg], RCS in dust [ng/mg], PGN in dust [ng/mg], GLU in dust [ng/mg], aerobic bacteria in dust [CFU/mg], anaerobic bacteria in dust [CFU/mg], and fungi in dust [CFU/mg]. The stimulants were the concentration levels of cytokines: IL-6 released by A549 and BEAS-2B cells [pg/mL], IL-8 released by A549 and BEAS-2B cells [pg/mL], and MTT IC50—mean dust concentration for A549 and BEAS-2B [mg/mL]. The analysis did not consider the variable “END in dust [ng/mg]”, since no differences between the sampling sites were found for this parameter.

The results of this analysis showed that the lowest value of the synthetic variable (1) referred to organic dust samples collected at the composting plant, thus indicating the highest potential health hazard in this facility. Conversely, the highest value of the synthetic variable (6) was observed for samples collected at the biomass-fired power plant, suggesting the lowest potential health impact from inhalable organic dust exposure at this plant.

## 3. Discussion

The present study has demonstrated that the commonly used term “organic dust” provides an imprecise description of the actual exposure conditions encountered in the workplace. Dust samples collected in various occupational environments varied with respect to particle surface morphology, chemical composition, and range of micro-biological components, as well as effects on human respiratory cells.

In our study, the strategy we adopted for the sampling and measurement of organic dust, based on using the filtration method during a single work shift, was associated with a risk of collecting insufficient dust mass for multiple laboratory analyses. The material usually used in similar studies is the settled dust [25,26,27,28,35]. However, as shown by O’Brian et al. [38], the bacterial profile of settled dust samples collected at a poultry farm differed significantly from that of inhalable dust samples. Therefore, they postulated that separate in vitro toxicological studies should be conducted in the future to distinguish between the immunomodulatory effects of exposure to microorganisms present in these two types of dust. The presumption about this difference was supported by the findings of a study on a small number of dust samples from a waste incineration plant. They showed that in airborne dust samples, endotoxin concentrations were nearly three times as high, and IL-8 released by A549 cells was twice as high as the respective values found for settled dust samples [35].

**Figure 6:**
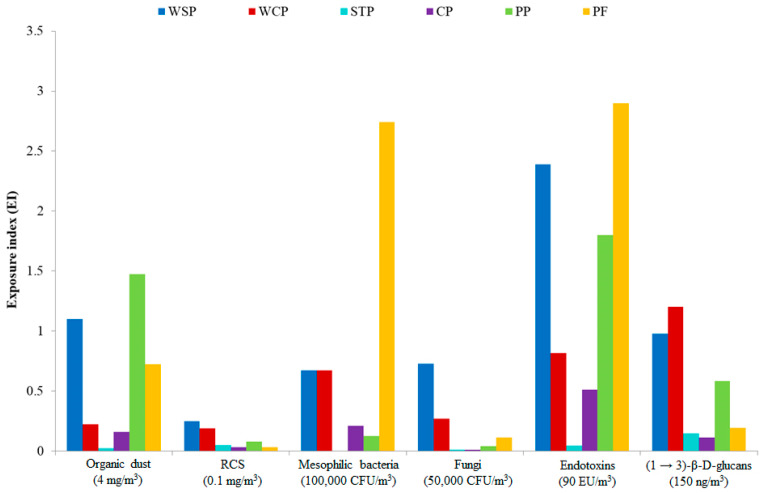
Assessment of exposure to organic dust components, based on exposure index, with available TLVs as reference values. WSP—waste sorting plant; WCP—waste composting plant; STP—sewage treatment plant; CP—cement plant; PP—power plant; PF—poultry farm; RCS—respirable crystalline silica.

In the present study, exposure to organic dust was measured in different workplaces during a single work shift. This strategy enabled assessing the potential health hazard by referring to available threshold limit values (TLVs). Figure 6 presents mean exposure levels for inhalable dust, RCS, mesophilic bacteria (aerobic and anaerobic), fungi, endotoxins, and (1 → 3)-β-D-glucans, in terms of exposure index (EI), where the reference points are the TLVs available in the literature (marked as index value “1”). In particular, TLVs were used as follows: inhalable dust—4 mg/m^3^; RCS—0.1 mg/m^3^; mesophilic bacteria—100,000 CFU/m^3^; fungi—50,000 CFU/m^3^ [39]; END—9 ng/m^3^ [40]; GLU—150 ng/m^3^ [41].

The calculations indicated that for most of the airborne agents present in the facilities under study, the EI values were below “1,” except for END concentrations at the WSP and PF, which were twice as high as the limit value proposed by NEG and DECOS [40]. Equally high EI values were found for mesophilic bacteria determined at the PF. Furthermore, the TLVs were also exceeded for inhalable dust concentrations at the WSP and PP, as well as for GLU at the WCP.

Exposure to bacteria, fungi, END and GLU has usually been assessed similarly to each other. In the present study, the concentrations of these agents did not differ significantly from the literature data for the following workplaces: WSP [42,43,44], WCP [6,45,46], STP [11,47,48], CP [49,50], PP [6,51,52], PF [53,54,55]. However, we would like to stress that, to the best of our knowledge, our findings regarding exposure to anaerobic bacteria at workstations in the WCP, CP, PP, and PF, as well as those on GLU in the CP, seem to be unique. The same refers to exposure to PGN, the concentrations of which have so far been assessed only for workstations at the WSP, WCP, and STP [11,56,57]. Reports on exposure to RCS in organic dust are relatively scarce. Only a few studies describe RCS concentration in the PF environment [58,59], in the STP [60], during biomass combustion [61], and in the CP [62].

The correlation coefficients that we obtained confirmed the findings of previous research, which pointed to inhalable organic dust as a good carrier of END. The limited number of samples may have contributed to the lack of significant positive correlations between the concentrations of inhalable organic dust and its microbial components [63]. However, RCS quantification indicates that RCS content can help explain some biological processes within the organic dust. In our study, RCS concentrations correlated with PGN, which is a component of the bacterial cell wall. This implies that bacterial cells may adhere to the surface of silicon particles [64,65]. Our findings did not show any correlation between the number of bacteria (aerobic and anaerobic) and RCS concentration. However, such a correlation was observed with PGN, which serves as an indicator of bacterial contamination. Interestingly, a moderate correlation between RCS and fungal concentrations may indicate, among other things, a possible biotransformation of amorphous silicon into SiO_2_ nanoparticles, which may involve the participation of certain fungi [66,67]. The coexistence of RCS and GLU, which is an indicator of fungal contamination, also supports this thesis.

A cytotoxicity study using the MTT assay demonstrated that bronchial epithelial cells were more sensitive to organic dust than type II alveolar cells, leading to reduced viability. The obtained IC50 values, at levels not exceeding 2 mg/mL, should be considered as very low. It is difficult to compare them with values reported in other studies, as the literature is scarce. One such study was conducted by Shen et al. [36], who were investigating the PM2.5 fraction in poultry houses. Using the MTT assay, they calculated an IC50 of 2.38 mg/mL, which was significantly higher than the currently reported IC50 for the samples from a poultry house (IC50 = 0.90 mg/mL). These differences may have been associated with differences in the particle sizes of the organic dust analyzed. To date, there is a lack of precise data regarding the effects that particular dust fractions may have on cytotoxicity. The decrease in cell viability that we observed in the present study is consistent with the findings of Monn and Becker [68] and Schins et al. [69], who reported that the PM10-2.5 fraction exhibited stronger cytotoxicity than the PM2.5 fraction. A different view was expressed by Cao et al. [70], who claimed that ultrafine and fine particles showed higher biological toxicity than the coarse particles. Another study by Viegas et al. [29] described an assessment of exposure to microbial contaminants in waste sorting facilities in Norway. However, it was impossible to compare our findings with these results because the authors used a quasi-qualitative method for cytotoxicity assessment and did not report the IC50 values they obtained.

Toxicological studies based on the analysis of settled dust provided considerably more data [25,27,28]. The inhibitory concentrations of settled organic dust after a 72 h exposure of A549 cells were several times higher than those found in our samples, indicating that settled dust may be less toxic than inhalable dust. However, the studies cited above showed that the highest cytotoxic potential was observed with organic dust exposure during sewage sludge management at the STP. In contrast, the settled organic dust collected at a poultry farm was the least toxic. As demonstrated by Szulc et al. [27] and Jabłońska-Trypuć et al. [71], sewage sludge contains numerous inorganic and organic compounds, including heavy metals and aromatic hydrocarbons. The EDS detection applied in our study revealed the presence of some elements on the surface of organic dust particles that could give rise to various chemical compounds. Salana and Verma [72], in their study on aerosol toxicity, demonstrated in vitro that the chemical composition of aerosol particles can significantly affect cellular viability.

The present study showed acceptable concentrations of inhalable organic dust in the workplace; however, the micro-biological contamination of the samples was high. This may have contributed to the inhibition of the test cell growth. Among the components of organic dust, END seems to be most responsible for the strong cytotoxic effect on the respiratory cells. However, the IC50 value for END depends on the particular bacterial species that are exposure agents. This value can range from ~0.2 µg/mL for *Escherichia coli* to 0.7 µg/mL for *Pseudomonas aeruginosa* [73]. Furthermore, the inhibitory concentrations for GLU are estimated at approx. 80 µg/mL [74], and for PGN at approximately 50 µg/mL [75]. Attempts have also been made to assess the cytotoxicity of some bacterial species, such as *P. aeruginosa* (IC50 ≈ 118 µg/mL) [76], or fungi, such as *A. fumigatus* (IC50 ≈ 200 µg/mL) and *A. terreus* (IC50 ≈ 500 µg/mL) [77]. This list of potential cytotoxic agents should be supplemented with RCS found in inhalable organic dust samples, with an IC50 value of ≈50 µg/mL [78], as well as all kinds of fungal metabolites and mycotoxins [79,80], and the novel components of organic dust, such as microplastic particles, which were detected during municipal waste management [81]. We presume that the combined effects of the individual components account for the differences in cytotoxicity among inhalable organic dust samples from the facilities we examined. However, the statistical analysis of our results, which was one of the few ever conducted for these parameters, demonstrated that the toxic effects of organic dust on human respiratory cells were similar across some occupational settings, namely WCP, STP, CP vs. WSP, PP, PF. This finding indicates a necessity for further studies to compare results from different workplaces.

The analytical findings confirmed that organic dust exposure not only reduces cell viability, but it can also stimulate the cells to secrete various chemokines and cytokines, which may induce an inflammatory process. In particular, the cytokine secretion profile for the immortalized human bronchial epithelial cell line BEAS-2B exposed to organic dust extracts may provide insight into the potential role of organic dust components in inflammation-induced respiratory diseases. Our study revealed a significant correlation: the intensity of IL-6 secretion from BEAS-2B cells increased with the increasing IC50 values, i.e., with the decreasing levels of dust cytotoxicity. Furthermore, GLM analysis demonstrated that the inhalable dust concentration had the most substantial impact on this cytokine’s concentration. These outcomes indicate that the respiratory epithelial cells exposed to organic dust particles initially respond by releasing inflammatory mediators, which continues until a certain level of dust concentration is exceeded. This triggers mechanisms that eventually lead to cell death. The cellular response to acute exposure to organic dust has been the subject of numerous studies, and some authors postulate that the oxidative stress induced by dust’s chemical components may be a causative factor in cell necrosis [82].

Our study demonstrated that the bronchial and alveolar epithelial cells exhibited a limited and uneven cytokine response to inhalable organic dust. The respiratory epithelium acts as a barrier between the external environment and the body’s interior. Its disruption triggers activation of the defense mechanisms through the production and release of proinflammatory agents, including cytokines and chemokines. These, in turn, activate the influx of inflammatory cells, such as neutrophils, eosinophils, natural killer (NK) cells, and macrophages, into cells exposed to harmful agents, such as bacteria or fungi. Exposure of test cells to aqueous dust extracts resulted in the release primarily of IL-6 and IL-8 cytokines. Interleukin 6 is a proinflammatory cytokine induced by environmental factors, and plays a crucial role in the development of community-acquired pneumonia [83]. Macrophages and monocytes produce interleukin 6, activate T lymphocytes, and regulate the growth and differentiation of B lymphocytes. Interleukin 8 is released from monocytes and endothelial cells in response to the increasing concentrations of IL-1β, TNFα, and reactive oxygen species. IL-8 is a chemotactic factor that induces neutrophil influx to the sites of dust deposition and inflammatory response.

In our experiment, low concentrations of TNF-α and IL-1β were observed. This can be attributed to differences in the kinetics of their release upon contact with organic dust. Regarding TNF-α, the highest blood concentrations were observed at approx. 6 h after exposure, and for IL-1β, at approx. 9–12 h after exposure [84], while the highest dynamics of IL-6 and IL-8 release occurred within 24–48 h after exposure and depended on the size of dust fraction [85]. The possible effect of particle size on the immunological response of respiratory cells appears to be an essential factor, as indicated by the findings of other studies [68,86]. Considering the above, it is easier for us to understand the differences between our analysis results and those reported by Shen et al. [36], who examined the PM2.5 fraction of dust samples collected at a poultry farm. They reported an IL-6 concentration of 15 pg/mL, whereas in the present study we found a value as high as 1180 pg/mL. For IL-8, the respective values were 25 pg/mL vs. 4300 pg/mL.

According to the literature, the concentration of cytokines released in vitro may also depend on the cell lines used in the assay. The IL-1β concentrations reported by Viegas et al. [30] were significantly higher than those found in our study, most likely because they used the THP-1 monocyte line. Interestingly, in our research, cytokine secretion was detected only for the BEAS-2B cells. This finding is consistent with the results of studies involving *Pseudomonas aeruginosa* strains [87]. Furthermore, it has been demonstrated that A549 cells are not effective producers of TNF-α [88], and this finding has been confirmed by Wang et al. [89] in their investigation of the immunotoxic properties of swine dust.

In our study, the multivariate statistical analysis using GLM shed new light on RCS and the numerous microbial determinants that can induce a release of proinflammatory cytokines from respiratory epithelial cells. Our findings pointed to the risk of significant underestimation when examining only single exposure agents, whose impact on cellular biological processes may differ when other organic dust components are also considered in the assessment. It is worth noting that END, the strongest immunomodulator, has currently been analyzed in numerous studies. In our research, it was found to significantly stimulate IL-8 secretion of when BEAS-2B cells were exposed.

The findings of our study can be regarded as a specific extension of the conclusions formulated, among others, by Palmberg et al. [90] and Redente and Massengale [91], who noted that IL-8 concentrations did not always correlate with END concentrations in organic dust. Statistical analysis (Table 7) showed that the BEAS-2B response to END, PGN, and GLU exposure would not proceed as expected in all cases. END contributed to the increase in IL-8 concentrations, while PGN and GLU acted as cytokine inhibitors. With regard to the PGN content in inhalable organic dust samples, the outcomes of the present study confirmed our earlier findings from the analysis of nasal lavage fluid from workers at waste sorting and sewage treatment plants [11]. However, the results regarding GLU did not support the hypothesis that END exerts a synergistic effect on the respiratory cells [92]. Instead, GLU demonstrated an immunomodulatory effect, manifested by reduced secretion of proinflammatory cytokines [93,94].

The GLM analysis revealed that the presence of RCS in organic dust had significantly contributed to the increase in IL-8 concentrations released by BEAS-2B cells. This highlights the need to control RCS concentration in organic dust, as other studies have indicated its immunotoxic potential [95]. Moreover, the effect of RCS on bronchial epithelial cells was found to be stimulated by the presence of fungi in organic dust. Previous studies reported that molds, such as *Aspergillus fumigatus*, can enhance the proinflammatory properties of silicon [96] by the secreting of IL-1β.

The effect that the fungal content in organic dust may have on particular cell lines may vary between species, and it may also depend on the part of the fungal structure that comes into contact with the respiratory cells [97]. In our study, the somewhat ambiguous results of the GLM analysis may have been due to the varying qualitative composition of the mycobiota present in the occupational environments under study. They can also be attributed to the presence of various fungal metabolites and mycotoxins, which, however, were not considered in our analysis. The available reports on some of these agents have shown that they can significantly stimulate respiratory cells to secrete various inflammatory mediators, including IL-6 and IL-8 cytokines [98,99].

The immunotoxic properties of mycotoxins could be enhanced by the presence of other microorganisms, particularly bacteria [100]. The high bacterial contamination of the dust samples in our study was also crucial when we analyzed the determinants of proinflammatory mediator release by the test cells. The aerobic bacteria contributed to decreased IL-6 and IL-8 levels. Therefore, our results highlight the need to consider aerobic bacteria in assessing workplace exposure to biological agents. The proinflammatory effect of bacteria, as noted for fungi, could have been modified by specific bacterial species (which we did not analyze) and their toxins, which have immunotoxic potential [101].

It should be noted that the present study was conducted during the SARS-CoV-2 pandemic. Therefore, we cannot exclude the possibility that some coronavirus particles could also be present in organic dust samples collected at the STPs and WSPs. It has been demonstrated that both sewage [102] and municipal waste [103] can be contaminated with this virus, which may have triggered proinflammatory processes in the respiratory systems of exposed workers [104].

Regarding the exposure to different components of inhalable organic dust and their effects on respiratory epithelial cells, our findings unveiled a complex picture of the threats these agents pose to human health. Factors such as the complex structure of organic dust particles, as well as the chemical and biological composition of organic dust have influenced cell lines at multiple levels. This finding made it particularly difficult to estimate the potential health hazard from exposure to inhalable organic dust. In view of the above, we decided to use a statistical method and establish a synthetic measure that could incorporate most of the study variables and allow us to order the workplaces we studied by the potential health hazard posed by organic dust exposure. This approach has previously been applied in various environmental studies [105,106], and in a recent project on occupational exposure to bioaerosols [107]. However, to the authors’ best knowledge, synthetic measures have not been used in in vitro respiratory cell models. In the present study, the variables described in Section 2 were used as a reference for calculating synthetic variables. Their values showed that the highest risk of respiratory effects from exposure to organic dust was associated with working conditions at the waste composting plant. At this point, it should be highlighted that single-shift sampling under pandemic constraints limited seasonal and day-to-day representativeness and might bias the presented hazard ranking. However, the presented methodological approach is open-ended, which allows for an unlimited number of variables to be included in the model. Should we have access to more precise data on the hazards identified through our analyses (e.g., mycotoxins, other chemical components, etc.), or to different cell lines for a comparative study, we could consider them in these calculations. Such a multifactorial approach would provide more substantial grounds for a conclusion about the adverse health effects of organic dust exposure for workers in different occupational settings. This method is a good option for assessing and comparing health risks among individuals exposed to organic dusts.

## 4. Materials and Methods

### 4.1. Sampling Sites

A field study was conducted between August 2020 and March 2021 in 6 enterprises; however, due to the SARS-CoV-2 pandemic, access to some of them was restricted. Detailed characteristics of the study facilities are presented in Table 9.

### 4.2. Sampling Strategy

Measurements of inhalable dust concentrations, RCS, viable bacterial and fungal microorganisms, PGN, END, and GLU were carried out at selected measuring points (Table 9) using CIS samplers (Conical Inhalable Sampler, Casella Measurements Inc., Bedford, UK), which can effectively capture particles with the size range of 6–60 µm, with an efficiency of 100–50%, respectively [108]. Each head was connected to a pump with a set flow of 3.5 L/min (APEX model, Casella Measurements Inc., UK). Measurement time was approximately 6 h. Since three kinds of samples were collected, the CIS samplers were loaded with the following three types of membrane filters (37 mm dia.).

- Polyvinyl chloride filters (PVC, SKC Inc., Eighty Four, PA, USA), with 5 µm pores, were used for dust collection and determination of RCS content.
- Teflon filters (PTFE, SKC Inc., USA), with 2 µm pores, were used for dust collection and immunotoxicity testing of organic dust; after the measurement, the filters were weighed and frozen at −20 °C.
- Glass fiber filters (GF/A, Whatman Inc., Kent, UK) for PGN, END, and GLU determination and to assess bacterial (aerobic and anaerobic) and fungal concentrations. After the measurement, each filter was divided in half, with one part used to determine PGN, END, and GLU concentrations, and the other for microbial analysis. In view of the potential micro-biological contamination of GF/A filters, each was depyrogenised at high temperature (180 °C) for 3 h. Moreover, all the CIS samplers were washed in a 1% E-Toxa-Clean solution (Sigma-Aldrich, Poznań, Poland). In this way, a total of 78 inhalable organic dust samples were collected for analysis.

A gravimetric method was used to assess organic dust concentration. Each filter was weighed in the laboratory before and after the measurements, using an XS-105DU balance (Mettler-Toledo GmbH, Greifensee, Switzerland), with instrument accuracy of 0.01 mg. Before weighing, the filters were conditioned for 24 h at controlled microclimate conditions (average temperature 21.6 °C, standard deviation (SD) 1.70; average humidity 41.8%, SD 6.05).

### 4.3. Organic Dust Surface Morphology

The surface morphology of organic dust particles was examined using a scanning electron microscope with cold field emission, model SU8010 (Hitachi, Tokyo, Japan). To enhance conductivity, the specimens were coated with a platinum/palladium layer using an ion beam sputter coater, Q150T ES, Quorum Technologies. Observations were conducted with an accelerating voltage of 10.0 kV and magnifications ranging from 500 to 5000, with a working distance of 8.6–15.7 mm.

Elemental analysis of dust particle surface and of carbon deposits was performed using Thermo Scientific NORAN System 7 with an electrically cooled Silicon Drift Detector (SDD) EDS (Thermo Scientific UltraDry, Waltham, MA, USA). Specimens were fixed on carbon tapes and coated with a conductive layer to make the characteristic platinum and palladium radiation lines to be visible in the EDS spectra. These peaks were not considered in the assessment of the chemical composition of the specimens. The results are presented in a table format, showing the elements identified on organic dust surface.

### 4.4. RCS Content Assessment

The study was conducted in an accredited laboratory, in compliance with the provisions of ISO 19087 [109]. Crystalline silica (quartz and cristobalite) content in inhalable fraction was determined. However, the analytical technique used (Fourier transform infrared spectroscopy, (FTIR)) is specific to the respirable dust fraction. Therefore, to reduce measurement uncertainty due to light scattering, mineralized samples were ground in an agate mortar for 5 min to decrease dust grain diameter to 10 µm or less. The percentage of RCS in the sample was then converted to mg/m^3^ air and µg/mg dust.

### 4.5. Assessment of Microbial Contamination

Half of each GF/A filter was eluted in 5 mL of PBS, and then 0.1 mL of the suspension was plated onto micro-biological media to identify the following groups of microorganisms: aerobic bacteria (Trypcase Soy Agar with 5% addition of defibrinated sheep blood), anaerobic bacteria (Schaedler Agar with 5% addition of defibrinated sheep blood) and fungi (Malt Extract Agar (MEA)).

The incubation conditions were as follows: aerobic bacteria—1 day, 37 °C; anaerobic bacteria—2 days, 37 °C; fungi—4 days, 30 °C. After the incubation period, the number of colony-forming units (CFUs) on the micro-biological medium was calculated and presented in CFU/m^3^ of air and CFU/mg of dust.

### 4.6. Determination of PGU, END, and GLU

For PGN, END, and GLU analysis, half of each GF/A filter was eluted in 5 mL of LRW (LAL Reagent Water) (Lonza Inc., Walkersville, MD, USA) with 0.05% Tween 20 (Sigma, Poland). The samples were eluted on a platform shaker for 15 min and then centrifuged at 1000× *g* for 15 min. The supernatant was used for both END and PGN analysis. In a further step, NaOH was added to the remaining supernatant to obtain a 0.3 M solution. Thus, prepared samples were eluted on a platform shaker at 4 °C for 10 min, centrifuged at 1000× *g* for 15 min, and used for GLU analysis.

PGN determination was carried out using the kinetic version of the SLP (Silkworm Larvae Plasma) test (Wako Pure Chemical Industries, Ltd., Osaka, Japan). After equal amounts (30 µL) of dust samples and the SLP lysate were added to each well, PGN concentrations were determined by spectrophotometry with Sunrise reader (Tecan Group Ltd., Männedorf, Switzerland) at 650 nm wavelength and at 30 °C. The concentration values were presented as ng/m^3^ air and ng/mg dust.

END was determined using the kinetic chromogenic (Kinetic-QCL) version of the LAL (*Limulus* Amebocyte Lysate) test (Lonza Inc., Walkersville, MD, USA). After equal amounts (100 µL) of dust samples and the LAL lysate were added to each well, END concentrations were determined using a microplate reader according to LAL assay requirements, at 405 nm and 37 °C. Standard endotoxin (CSE) activity was 11 EU/ng. The concentration values were presented as ng/m^3^ air and ng/mg dust.

GLU analysis was performed using the kinetic version of the Glucatell assay (Associates of Cape Cod Inc., East Falmouth, MA, USA). After 25 µL of dust sample and 50 µL of reagent were added to each well, GLU concentration was determined by a spectrophotometer with a microplate reader, at 405 nm and 37 °C according to requirements of the Glucatell test. The concentration values were presented as ng/m^3^ air and ng/mg dust.

### 4.7. Cell Cultures

The studies were conducted on human lung cancer cell line A549, obtained from the Technical University of Berlin, and on bronchial epithelial cells BEAS-2B, from the ATCC collection (CRL-9609, LGC Standards Ltd., Łomianki, Poland). A549 cells were cultured in complete DMEM medium with 10% FBS and 1% antibiotics and antimycotic agents. BEAS-2B cells were cultured in serum-free LHC-9 medium (Life Technologies Ltd., Carlsbad, CA, USA) with supplemented antibiotics, including penicillin (100 U/mL) and streptomycin (100 μg/mL) (Life Technologies Ltd., USA), at the medium concentration of 1 mL/100 mL. The culture was incubated under sterile conditions (37 °C; 5% CO_2_; 95% humidity) in culture vessels with a culture growth surface of 25 cm^2^ (Nalge Nunc International, Rochester, NY, USA).

Prior to toxicity testing, a cell suspension was prepared. Cells were dissociated using trypsin (0.25% trypsin with EDTA), then resuspended in fresh culture medium and centrifuged (200× *g* for 5 min) to remove the cells damaged by trypsinization. Cell viability was assessed using Trypan blue exclusion assay.

### 4.8. Cytotoxicity Assessment

The cytotoxic effect of the tested organic dust was analyzed using MTT tetrazolium salt reduction assay (MTT test), which measures mitochondrial dehydrogenase activity in living cells. Cells were suspended in culture medium at a concentration of 1.0 × 10^5^ viable cells/mL (A549) or 1.5 × 10^5^ viable cells/mL (BEAS-2B) and then plated onto 96-well microplates. The tested dusts were suspended in a culture medium appropriate for a given cell line. After 24 h of incubation, the medium was aspirated, and suspensions of dust samples at a specified concentration were added, (9 repetitions for each microplate). Two or three independent experiments were performed, depending on the amount of dust in the sample. After another 24 h, colorimetry was carried out (λ = 570/620 nm) using a Synergy 2 microplate reader (BioTek Instruments Inc., Winooski, VT, USA). Toxicity was calculated as a ratio of absorbencies of the treated and untreated cells. The concentration values that induced a 20% (IC20 index) and 50% (IC50 index) decrease in cell viability for each dust sample were extrapolated using linear regression for the average of 7 middle values from 9 replicates (min. and max. values were discarded to avoid overfitting and outliers) according to the concentration level (mg/mL). The linear regression based on 7 middle observations were estimated for each dust sample. Using regression parameters, the theoretical (extrapolated) value for 20% and 50% was calculated. The linear extrapolation goodness of fit was very good, R^2^ = [0.8–0.96].

### 4.9. Determination of Proinflammatory Cytokines

After 24 h of incubation of A549 and BEAS-2B cells (37 °C, 5% CO_2_, 95% humidity), the supernatant was aspirated and replaced with medium containing a suspension of the tested dusts at a specified concentration. Then, after 24 h of exposure, the supernatant was collected, centrifuged, and a protein cocktail containing protease inhibitors was added before freezing the supernatant in liquid nitrogen. Cytokines (IL-1β, IL-6, IL-8, and TNF-α) released from the tested cells were determined using Quantikine ELISA kits (R&D Systems Inc., Minneapolis, MN, USA) according to manufacturer’s protocol. The assays had the following detection levels: IL-1β, 0.58 pg/mL; IL-6, 0.54 pg/mL; IL-8, 2.76 pg/mL; and TNF-α, 0.45 pg/mL.

### 4.10. Statistical Analysis

Descriptive statistics for exposure and immunotoxicity assays included calculations of the arithmetic mean, standard deviation, median value, and range of results. Correlation analysis using Pearson’s linear correlation coefficient was also performed. ANOVA with Welch’s correction was applied to assess the significance of differences between group means, and the Benferroni *t*-test was used for pairwise comparisons. The relationship between cytokine secretion and exposure agents was evaluated using a generalized linear model (GLM).

The data on organic dust exposure, cytotoxicity, and cytokine secretion were used to rank inhalable organic dust sources by the health hazard they pose. A taxonomic linear ordering method was applied, based on the concept of an unobservable variable whose values are estimated from observable variables that describe the objects under study [37]. According to the linear ordering methodology, the first step was to determine the model object, based on the matrix of standardized output variables, with the minimum for destimulants and the maximum for stimulants. Then, the distance from the model object was determined using the Euclidean distance (*d*_*i*0_). Finally, the synthetic variable was calculated according to the following formula:

$$s_{i}=1-\frac{d_{i0}}{d_{0}},i=1,2,\ldots ,m,$$

where

$$d_{0}=\bar{d_{0}}+2S\left(d_{0}\right),$$

whereby

$$\bar{d_{0}}=\frac{1}{n}\sum_{i=1}^{n}d_{i0};S\left(d_{0}\right)={\left[\frac{1}{n}\sum_{i=1}^{n}{\left(d_{i0}-\bar{d_{0}}\right)}^{2}\right]}^{\frac{1}{2}}$$

The synthetic measure assumes values within the range of [0; 1]. The values become higher the closer an object is to the model object. Based on the analyzed data, this means that in the given facility under study, the potential health hazard from workers’ exposure to organic dust decreased with increasing values of the aggregated variable. All analyses were performed using SAS 9.4 software (SAS Institute Inc., Cary, NC, USA). The *p*-value of 0.05 was adopted as the level of significance in all the tests performed.

## 5. Conclusions

Our study appears to be one of the few to confirm that inhalable organic dust is characterized by a diversity of chemical and micro-biological components, depending on the environment in which it occurs. Organic dust exposure was found to have a significant cytotoxic effect on human respiratory cells and the intensity of proinflammatory cytokine release. By applying multivariate statistical analysis, we were able to precisely identify those exposure agents that had a significant impact on the functions of respiratory epithelial cells. We found that the most hazardous agents include respirable crystalline silica, aerobic bacteria, fungi, as well as peptidoglycans and (1 → 3)-β-D-glucans. They significantly affected cytokine release, particularly of IL-6 and IL-8. The use of a synthetic measure enabled assessment of the organic dust collected at the composting plant, which showed the highest potential to induce adverse effects on human health. This open-ended statistical method, which can be used both for exposure assessment and in vitro tests, is a promising tool for the identification and evaluation of the potential health risks associated with exposure across various occupational settings. It can significantly increase awareness of occupational hazards and, consequently, lead to more responsible protection of exposed workers.

## Figures and Tables

**Table 1:** Qualitative comparison of elements detected on organic dust particle surfaces by EDS analysis.

Element	WSP	WCP	STP	CP	PP	PF
C	+	+	+	+	+	+
O	+	+	+	+	+	+
N	+	+	+	+	+	+
Al	+		+	+	+	
Si	+		+	+	+	
F	+					
Na	+		+			
Mg	+		+			
S	+			+		
Ca	+	+	+	+		
P						+

WSP—waste sorting plant; WCP—waste composting plant; STP—sewage treatment plant; CP—cement plant; PP—power plant; PF—poultry farm; + —revealed presence of the element in the tested samples.

**Table 2:** Exposure agents in inhalable organic dust samples from workstations in facilities under study (AM (SD)).

Agent	WSP	WCP	STP	CP	PP	PF	ANOVA (F; *p*)
Inhalable organic dust (mg/m^3^)	4.41 (4.96)	0.89 (0.34)	0.09 (0.03)	0.64 (0.78)	5.90 (8.40)	2.90 (0.66)	**19.1; *p* < 0.001**
RCS (mg/m^3^)	0.025 (0.026)	0.019 (0.008)	0.005 (<0.001)	0.003 (0.001)	0.008 (0.013)	0.003 (<0.001)	**25.2; *p* < 0.001**
Aerobic bacteria (×10^3^ CFU/m^3^)	26.1 (7.37)	50.6 (24.6)	0.37 (0.13)	12.0 (19.6)	7.80 (14.2)	201 (62.7)	**16.9; *p* < 0.001**
Anaerobic bacteria (×10^3^ CFU/m^3^)	41.1 (26.7)	16.8 (6.70)	0.02 (0.04)	8.86 (16.1)	4.49 (8.37)	72.7 (13.5)	**23.7; *p* < 0.001**
Fungi (×10^3^ CFU/m^3^)	36.3 (27.9)	13.5 (7.46)	0.46 (0.68)	0.54 (0.65)	2.08 (2.83)	5.56 (3.68)	**4.53; *p* = 0.027**
END (ng/m^3^)	23.9 (21.5)	73.5 (33.2)	3.93 (2.80)	46.0 (67.5)	162 (248)	261 (163)	**5.90; *p* = 0.014**
PGN (ng/m^3^)	5918 (6765)	7930 (6046)	237.7 (90.3)	4089 (6301)	2693 (2422)	4486 (2531)	**4.18; *p* = 0.037**
GLU (ng/m^3^)	146 (78.5)	180 (46.4)	21.7 (8.65)	16.8 (19.7)	87.6 (52.5)	29.2 (31.1)	**10.9; *p* = 0.001**

AM—arithmetic mean; SD—standard deviation; WSP—waste sorting plant; WCP—waste composting plant; STP—sewage treatment plant; CP—cement plant; PP—power plant; PF—poultry farm; RCS—respirable crystalline silica; END—endotoxins; PGN—peptidoglycans; GLU—(1 → 3)-β-D-glucans.

**Table 3:** Pearson correlation coefficients for correlations between inhalable organic dust components.

Variable	(1)	(2)	(3)	(4)	(5)	(6)	(7)
(1) Inhalable organic dust	1.00						
(2) RCS	**0.64** (***p* < 0.001**)	1.00					
(3) Aerobic bacteria	0.11 (*p* = 0.583)	−0.12 (*p* = 0.594)	1.00				
(4) Anaerobic bacteria	0.23 (*p* = 0.259)	0.05 (*p* = 0.795)	**0.82** (***p* < 0.001**)	1.00			
(5) Fungi	0.36 (*p* = 0.073)	**0.68 ** (***p* < 0.001**)	0.03 (*p* = 0.881)	**0.44** (***p* = 0.027**)	1.00		
(6) END	**0.62** (***p* < 0.001**)	0.34 (*p* = 0.089)	**0.45** (***p* = 0.024**)	**0.71** (***p* < 0.001**)	**0.60** (***p* = 0.001**)	1.00	
(7) PGN	0.30 (*p* = 0.143)	**0.54** (***p* = 0.005**)	0.21 (*p* = 0.306)	0.30 (*p* = 0.144)	**0.41** (***p* = 0.038**)	0.33 (*p* = 0.105)	1.00
(8) GLU	0.16 (*p* = 0.434)	**0.52** (***p* = 0.007**)	−0.12 (*p* = 0.567)	0.07 (*p* = 0.730)	**0.48** (***p* = 0.013**)	0.33 (*p* = 0.103)	0.32 (*p* = 0.112)

**Table 4:** Microbial agents and RCS in inhalable organic dust samples subject to immunotoxicity assessment (AM (SD)).

Agent	WSP	WCP	STP	CP	PP	PF	ANOVA (F; *p*)
Inhalable organic dust (mg)	4.11 (3.52)	0.98 (0.46)	0.07 (0.02)	0.46 (0.62)	7.31 (9.88)	2.04 (0.61)	**10.4; *p* = 0.002**
RCS (µg/mg)	9.89 (3.84)	22.3 (3.24)	57.5 (6.77)	9.32 (10.9)	8.28 (6.24)	0.89 (0.10)	**76.2; *p* < 0.001**
Aerobic bacteria (×10^3^ CFU/mg)	19.7 (11.9)	101 (52.5)	7.27 (2.66)	24.6 (21.8)	2.60 (1.40)	139 (18.1)	**39.5; *p* < 0.001**
Anaerobic bacteria (×10^3^ CFU/mg)	32.7 (37.2)	33.2 (13.9)	0.45 (0.90)	21.2 (14.9)	0.99 (0.92)	53.8 (17.0)	**12.8; *p* = 0.001**
Fungi (×10^3^ CFU/mg)	23.9 (31.0)	25.4 (14.8)	8.59 (12.2)	1.34 (1.42)	3.95 (6.99)	3.54 (1.83)	2.94; *p* = 0.082
END (ng/mg)	10.7 (15.3)	10.1 (3.16)	3.90 (3.02)	9.54 (12.2)	7.37 (9.48)	7.35 (4.32)	1.45; *p* = 0.297
PGN (ng/mg)	1359 (906)	9779 (6624)	2013 (657)	6735 (3784)	5477 (9563)	1584 (829)	2.77; *p* = 0.087
GLU (ng/mg)	78.5 (74.5)	242 (77.7)	191 (93.4)	37.9 (23.6)	122 (141)	13.8 (19.0)	**8.30; *p* = 0.004**

AM—arithmetic mean; SD—standard deviation; WSP—waste sorting plant; WCP—waste composting plant; STP—sewage treatment plant; CP—cement plant; PP—power plant; PF—poultry farm; RCS—respirable crystalline silica; END—endotoxins; PGN—peptidoglycans; GLU—(1 → 3)-β-D-glucans.

**Table 5:** Pearson correlation coefficients for the IC20 and IC50 values and cytokine concentrations.

Variable	A549		BEAS-2B		
	IL-6	IL-8	IL-1β	IL-6	IL-8
IC20	0.03 (*p* = 0.787)	−0.01 (*p* = 0.927)	−0.30 (*p* = 0.129)	0.02 (*p* = 0.918)	−0.22 (*p* = 0.587)
IC50	0.16 (*p* = 0.190)	−0.02 (*p* = 0.844)	0.19 (*p* = 0.332)	**0.87 (*p* < 0.001)**	0.27 (*p* = 0.181)

**Table 6:** Description of exposure agents affecting proinflammatory cytokine release by the A549 cell line, based on GLM analysis.

Agent	A549					
	IL-6			IL-8		
	Parameter	Chi-Square Wald	*p*-Value	Parameter	Chi-Square Wald	*p*-Value
Intercept	1456.669	119.67	***p* < 0.001**	8.8766	2864.52	***p* < 0.001**
Inhalable organic dust	425.3069	8.12	***p* = 0.004**	0.1276	0.44	*p* = 0.506
RCS	−6.1665	3.09	*p* = 0.079	−0.0090	4.39	***p* = 0.036**
Aerobic bacteria	−0.0030	11.64	***p* = 0.001**	−0.0000	11.19	***p* = 0.001**
Fungi	0.0126	9.66	***p* = 0.002**	0.0000	17.11	***p* < 0.001**
END	−1.1219	0.01	*p* = 0.906	−0.0071	0.38	*p* = 0.535
PGN	0.0106	1.00	*p* = 0.317	−0.0000	0.19	*p* = 0.659
GLU	−0.9556	2.34	*p* = 0.126	0.0011	1.67	*p* = 0.196

**Table 7:** Description of exposure agents affecting proinflammatory cytokine release by the BEAS-2B cell line, based on GLM analysis.

Agent	BEAS-2B								
	IL-1β			IL-6			IL-8		
	Parameter	Chi-Square Wald	*p*-Value	Parameter	Chi-Square Wald	*p*-Value	Parameter	Chi-Square Wald	*p*-Value
Intercept	2.0576	82.43	***p* < 0.001**	1393.7	106.06	***p* < 0.001**	8.4416	1301.14	***p* < 0.001**
Inhalable organic dust	1.3518	17.39	***p* < 0.001**	598.40	6.77	***p* = 0.009**	0.4863	1.63	*p* = 0.201
RCS	0.0098	3.62	*p* = 0.057	−5.3103	3.25	*p* = 0.071	0.0213	16.08	***p* < 0.001**
Aerobic bacteria	−0.0000	2.40	*p* = 0.121	−0.0033	12.52	***p* < 0.001**	−0.0000	11.08	***p* = 0.001**
Fungi	0.0000	0.34	*p* = 0.558	0.0130	8.99	***p* = 0.003**	−0.0000	7.02	***p* = 0.008**
END	−0.0375	2.09	*p* = 0.148	−2.0075	0.04	*p* = 0.841	0.0565	4.61	***p* = 0.032**
PGN	0.0001	1.45	*p* = 0.228	0.0160	2.42	*p* = 0.119	−0.0003	18.00	***p* < 0.001**
GLU	0.0005	0.40	*p* = 0.528	−0.9004	2.80	*p* = 0.094	−0.0024	6.99	***p* = 0.008**

**Table 8:** Potential health hazard from exposure to inhalable organic dust in particular facilities, as ranked with the use of taxonomic linear ordering [37].

Facility	Synthetic Variable	Ordering
WCP	0.099237	1
STP	0.147100	2
CP	0.247099	3
PF	0.343623	4
WSP	0.424320	5
PP	0.520128	6

**Table 9:** Characteristics of facilities designated for organic dust sampling.

Facility Symbol	Short Description of Facility	Sampling Points	Number of Samples
WSP	A mixed-waste sorting plant (60,000 tons/year).	Conveyor belt loading, preliminary sorting cabin, press for recyclable materials, bioreactors.	12
WCP	A green-waste composting plant (12,000 tons/year).	Compost sieve, bioreactors, green-waste shredders (2), green-waste piles.	15
STP	A municipal sewage treatment plant with sewage sludge incinerator (165,000 m^3^/day).	Conveyor belt for sewage sludge (2), sewage sludge dump chamber (2).	12
CP	A cement plant with refuse-derived fuel (RDF) used for clinker burning (85,000 tons/year).	RDF storage hall (3), RDF conveyor belt (2).	15
PP	A power plant with forest and agricultural biomass co-combustion (1.2 million tons/year).	Rehandling building, conveyor belt for biomass, biomass storage chamber, biomass hopper.	12
PF	A poultry farm with intensive broiler breeding, a henhouse with 25,000 chickens.	Poultry house (at a distance of 5 m, 15 m, 25 m, and 35 m from the entrance).	12

WSP—waste sorting plant; WCP—waste composting plant; STP—sewage treatment plant; CP—cement plant; PP—power plant; PF—poultry farm.

## Data Availability

The datasets supporting the conclusions of this article are available upon request from the corresponding author.

## Abbreviations

The following abbreviations are used in this manuscript:

HP	Hypersensitivity pneumonitis
ODTS	Organic dust toxic syndrome
COPD	Chronic obstructive pulmonary disease
RCS	Respirable crystalline silica
END	Endotoxins
PGN	Peptidoglycans
GLU	(1 → 3)-β-D-glucans
IL-1β	Interleukin 1 beta
IL-6	Interleukin 6
IL-8	Interleukin 8
TNF-α	tumor necrosis factor-alpha
LAL	*Limulus* Amebocyte Lysate
WSP	Waste sorting plant
STP	Sewage treatment plant
WCP	Waste composting plant
CP	Cement plant
PP	Power plant
PF	Poultry farm
GLM	Generalized linear model
